# Fatal strongyloidiasis after corticosteroid therapy for presumed chronic obstructive pulmonary disease

**DOI:** 10.1099/jmmcr.0.005165

**Published:** 2018-09-11

**Authors:** Priyatam Khadka, Pratap Khadka, Januka Thapaliya, Dhana Bikram Karkee

**Affiliations:** ^1^​ Medical Laboratory, Sumeru Hospital, Dhapakhel, Lalitpur, Nepal; ^2^​ Medical Microbiology, Tri-Chandra Multiple Campus, Tribhuvan University, Kathmandu, Nepal; ^3^​ Biotechnology, Tribhuvan University, Kathmandu, Nepal; ^4^​ Sumeru Hospital, Dhapakhel, Lalitpur, Nepal

**Keywords:** corticosteroid, hyper-infection, strongyloidiasis, immunosuppression

## Abstract

**Introduction:**

Strongyloidiasis is a neglected tropical disease with global prevalence. Under some cases of immune suppression (especially with corticosteroid administration), the nematode involved disseminates, leading to an amplified, possibly lethal hyper-infection syndrome.

**Case presentation:**

A 56-year-old Nepalese man presenting with chief complaints of nausea, vomiting, joint pain and abdominal cramps was admitted to Sumeru Hospital. His past history revealed: chronic obstructive pulmonary disease (COPD), systemic hypertension and previously treated pulmonary tuberculosis. The patient had been treated with oral prednisolone (60 mg gl^−1^) for 8 days due to a presumed exacerbation of his COPD. Sequentially, he developed haemoptysis, chest tightness, frequent wheezing and worsening cough. Bronchoscopy showed severe diffuse alveolar haemorrhage; microbiological examination of broncho-alveolar lavage (BAL) was recommended. Examination of an acid fast bacilli stain preparation of BAL revealed filariform larvae of *Strongyloides*. Stool specimen examination revealed larvae of *Strongyloides.* The physical condition of the patient began to deteriorate; a few days after admission, vancomycin-sensitive *Enterococcus faecium* was isolated from a blood sample. He was treated with ivermectin and albendazole for strongyloides and linezolid plus vancomycin for *E. faecium*. However, the patient failed to recover from the illness and died.

**Conclusion:**

The findings of our study suggest that corticosteroid administration in strongyloidiasis can lead to the development of fatal strongyloides hyper-infection syndrome. Hence our experience suggests the need for early diagnosis of strongyloidiasis to avoid such an outcome. A deterioration of the patient's condition after the initiation of corticosteroid therapy in endemic areas should raise the possibility of strongyloidiasis.

## Introduction

Strongyloidiasis is a neglected nematode infestation with an extensive global prevalence (especially in the tropics and sub-tropics). The global burden of strongyloidiasis is estimated to be between 30 and 100 million people [1]. In low-income countries, the infestation is commonly associated with those of lower socioeconomic backgrounds and with members of marginalized groups [2]. However, in the developed world, an association is seen with immunosuppression resulting from diseases such as lymphoma, leukaemia and AIDS or from the use of corticosteroids [3].

The rhabtidiform larvae of the nematodes mature to filariform larvae which penetrate the wall of the colon and amplify the infection, leading to strongyloides hyper-infection syndrome [4, 5]. Nearly half of all strongyloides infections remain asymptomatic and may persist for the life of the patient. A high index of suspicion is therefore required for early diagnosis and starting appropriate therapy. The onset of strongyloides hyper-infection syndrome is associated with a myriad of seemingly unrelated symptoms including diarrhoea, abdominal pain, urticaria, anaemia, sepsis (frequently with multiple organisms), shock and acute respiratory distress syndrome (ARDS) [6]. The mortality rate, nevertheless, approaches 90 % and thus diagnosis considerations are obligatory.

We here describe a patient who received steroid therapy due to a diagnosis of chronic obstructive pulmonary disease (COPD) and developed strongyloides hyper-infection syndrome and associated bacteraemia.

## Case presentation

A 56-year-old Nepalese man presenting with chief complaints of frequent wheezing, nausea, vomiting, joint pain and abdominal cramps was admitted to Sumeru Hospital on 15 November 2016. His past history revealed: COPD, systemic hypertension and formerly treated pulmonary tuberculosis. The patient had been under oral steroid therapy (prednisone 60 mg gl^−1^) 3 months previously, tapered to 5 mg with symptomatic improvement. Twelve days prior to presentation at Sumeru Hospital (3 November 2016), he was admitted to a local hospital with a diagnosis of acute gastritis with acute exacerbated COPD for 2 days. He was given intravenous ceftriaxone 2 g once daily, azithromycin 500 mg once daily, methylprednisone 40 mg three times daily, and salbutamol and ipratropium bromide nebulizer at that time. The prednisolone was given for a total of 8 days. Nonetheless, his pulmonary condition worsened with haemoptysis, chest tightness and increased cough.

On arrival at the emergency Intensive Care Unit, he was found to be hypotensive, hypoxaemic and febrile. Meanwhile, body temperature (37.78 °C), blood pressure (78/35 mmHg) and arterial partial pressure of oxygen (P_a_O_2_) (69 mmHg) were noted. Physical examination of the abdomen revealed epigastric tenderness but no hepatosplenomegaly. No oedema, cyanosis or clubbing was noted. Consequently, he was given a preliminary diagnosis of septic shock from an abdominal source and acute respiratory failure. Concurrently, mechanical ventilation, aggressive volume resuscitation and vasopressor support were rapidly begun. Piperacillin/tazobactam was administered empirically as an anti-infection treatment.

**Fig. 1.**
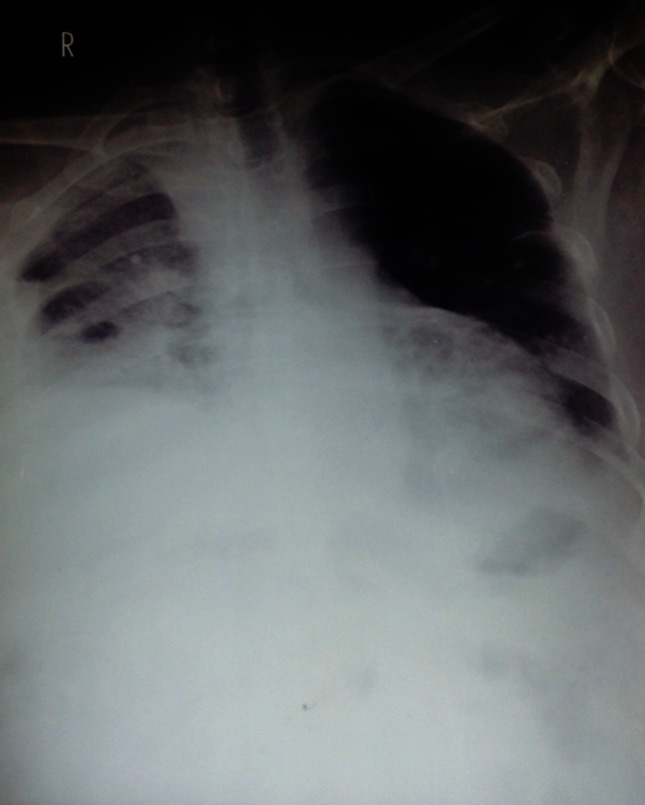
Chest X-ray: collapse consolidation with pleural effusion on the right lower lobe, hilar lymph nodes and cardiomegaly were noted.

On radiological assessment, chest X-ray showed collapse consolidation with pleural effusion on the right lower lobe, hilar lymph nodes and cardiomegaly (Fig. 1). Correspondingly, serology was negative for human immunodeficient virus (HIV), hepatitis B surface antigen (HBsAg) and hepatitis C virus (HCV); C-reactive protein had increased to 110 mg l^−1^ (normal, <10), white blood cell count to 16.5×10^9^ cells l^−1^ (normal, 4.0–10.0), with neutrophil percentage of 92 % (normal, 50–70 %) and four eosinophils counted. However, haemoglobin concentration, coagulation-related test, platelet count, renal function tests (RFTs) and liver function tests (LFTs) were within the normal range.

**Fig. 2.**
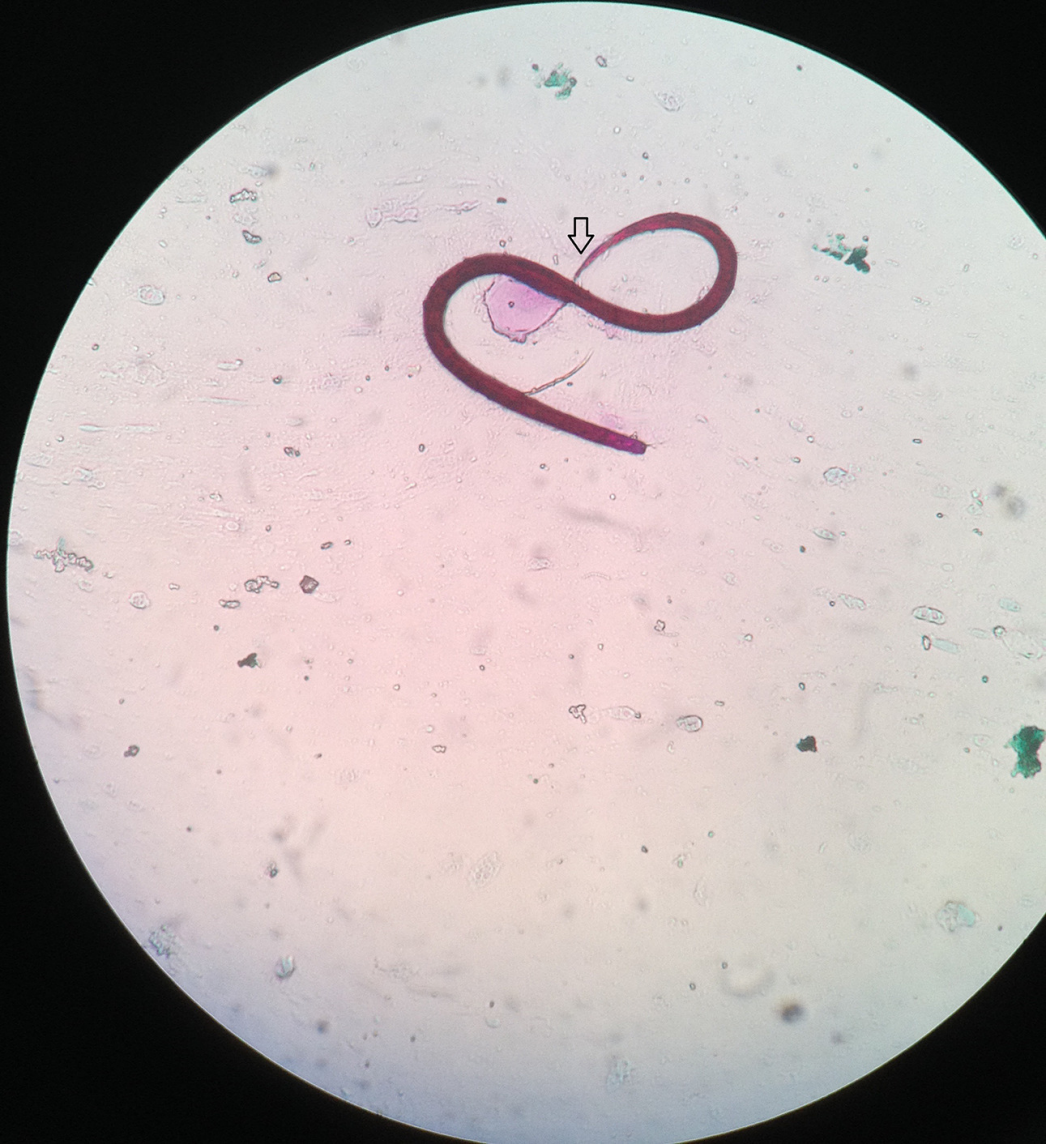
AFB staining*: Strongyloides stercoralis* [usual size: 0.9 mm (male); 2.0–2.5 mm (female)] in acid-fast stained broncho-alveolar lavage isolate (original magnification 400×).

A flexible bronchoscopy was performed on day 2 of admission; on bronchoscopy severe diffuse alveolar haemorrhage was seen. Therefore, microbiological examination of broncho-alveolar lavage (BAL) was recommended. Upon examination of an acid-fast bacilli (AFB) stain preparation of BAL, filariform larvae of *Strongyloides stercoralis* were seen but no AFB were found (Fig. 2). Neither fungal elements nor malignant cells were detected on subsequent fungal staining and cytological examination. With wet preparation of a stool specimen, numerous larvae of *S.stercoralis* were seen (Video S1 and Fig. S1, available in the online version of this article). Gradually, the physical condition of patient began to deteriorate and a few days after admission vancomycin-sensitive *Enterococcus faecium* was isolated from his blood sample. Therefore, treatment with piperacillin/tazobactam was stopped, and specific treatment was started for strongyloides hyper-infection syndrome.

He was then treated with ivermectin and albendazole for strongyloides with repeated daily stool examination to verify eradication and to exclude indwelling other parasitic infections. On day 1, the wet preparation of stool and sputum revealed actively motile larvae of *S. stercoralis*. After treatment with ivermectin (8000 µg po qd pc) and albendazole (400 mg po bid) larval counts reduced significantly to nil in the stool sample from day 4. Although the count reduced significantly, the species was found to be motile in sputum samples until day 5. The detailed treatment protocol, duration of treatment and parasite examinations of stool and sputum are shown in Table 1. Simultaneously, linezolid plus vancomycin was prescribed for two different strains of *E. faecium*.

**Table 1.** Anti-helmenthic activities on strongyloides hyper-infection syndrome

Day of treatment	Stool examination	Sputum examination	Ivermectin	Albendazole
1	Numerous larvae of *Strongyloides stercoralis* seen (actively motile)	Numerous *Strongyloides stercoralis* larvae seen (actively motile)	8000 µg po qd pc	400 mg po bid
2	Reduced number of *Strongyloides stercoralis* larvae (actively motile)	Reduced number of *Strongyloides stercoralis* larvae (actively motile)	8000 µg po qd pc	400 mg po bid
3	Reduced number of *Strongyloides stercoralis* larvae (sluggish)	Reduced number of *Strongyloides stercoralis* larvae (sluggish, <2/high-power field(40X)	8000 µg po qd pc	400 mg po bid
4	Not seen	Reduced number of *Strongyloides stercoralis* larvae (sluggish, <1/high-power field(40X)	8000 µg po qd pc	400 mg po bid
5	Not seen	Reduced number of *Strongyloides stercoralis* larvae (sluggish, <1/ high-power field(40X)	8000 µg po qd pc	400 mg po bid
6	Not seen	Not done	8000 µg po qd pc	400 mg po bid

On day 4 after admission, laboratory results revealed C-reactive protein level had doubled to 220 mg l^−1^. His white blood cell count had decreased to 2.9×10^9^ cells l^−1^ but eosinophil count was elevated to 10. Similarly, his haemoglobin concentration decreased to 48 g l^−1^ and platelet count was 7×10^9^ cells l^−1^. Furthermore, total bilirubin level increased to 103 mmol l^−1^. A brief blood investigation report is presented in Table 2.

**Table 2.** Blood investigation report on day 1, day 1 and the last day

Blood tests	Day 1	Day 2	Last day
Serological tests:			
(HIV, HBsAg, HCV), ELISA	Non-reactive		
CRP (mg l^−1^)	110	220	499
Haematological tests:			
WBC (10^9^ cells l^−1^)	16.5	2.9	6
Neutrophil percentage (%)	88	48	45
Eosinophils (10^9^ cells l^−1^)	0.04	0.1	0.18
H (g l^−1^)	140	48	55
Platelet count (10^9^ cells l^−1^)	160	7	13
INR 1.07 1.15	1.06	1.1	Interference by jaundice
PT (s)	13.6	13.3	Interference by jaundice
APTT (s)	24.2	33.7	Interference by jaundice
Fibrinogen level (g l^−1^)	2.3	2.7	1.9
d-Dimer (µg l^−1^)	2216	12 390	27 900
Biochemical tests:			
BUN (mmol l^−1^)	19	16	25
Creatinine (mmol l^−1^)	120	114	110
Albumin (g dl^−1^)	23	27	18
Total bilirubin (m mol L¯¹)	13	103	378
ALT (U l^−1^)	45	36	59
AST (U l^−1^)	31	90	78
Sodium (mmol l^−1^)	133	147	137
Potassium (mmol l^−1^)	3.9	4	3

ALT, alanine aminotransferase; APTT, active partial thromboplastin time; AST, aspartate aminotransferase; BUN, blood urea nitrogen; Hb, haemoglobin concentration; INR, international normalized ratio; PT, prothrombin time; WBC, white blood cell; HIV, human immunodefeciency virus; HBsAg, hepatitis B surface antigen; HCV, hepatitis C virus; CRP, C-reactive protein.

Although the number of larvae was dramatically reduced, the patient developed a high-grade fever, vomiting, lower abdominal pain, abdominal distention and constipation, dyspnoea, wheezing and pleuric pain, and ARDS. He was therefore mechanically ventilated. However, his condition worsened and he died on day 6 after admission. The results of blood tests on the last day are presented in Table 2. Brief details from the patient’s history and diagnostic approaches are organized as a timeline in Fig. S2.

## Discussion

Strongyloidiasis is a significant public health problem with varying degree of severity and clinical presentation. The infection is categorized into three types: acute, chronic uncomplicated, and chronic complicated or hyper-infection syndrome [7]. The hyper-infection syndrome is estimated to occur in 1.5–2.5 % of patients with strongyloidiasis; mortality can be up to 90 % [8].

Hyper-infection syndrome progresses when immunosuppression decreases the effectiveness of the immune system and results in augmentation of the normal life cycle of the parasite leading to a dramatic upsurge in larval density [9]. The rhabtidiform larvae mature into filariform larvae and penetrate the colonic mucosa of the host, carrying enteric organisms along with them. As part of their normal life cycle, they enter the lungs where they cause eosinophilic pneumonitis and what often appears at first to be an exacerbation of COPD, and which in turn may progress rapidly to ARDS [10–13].

The administration of corticosteroids has long been a mainstay of therapy for the treatment of acute exacerbations of COPD [14]. Short courses of corticosteroids (prednisone) have been shown to improve both spirometric and clinical outcomes in acute exacerbations of COPD [15]. On the one hand, corticosteroids (prednisone) decrease the risk for relapse by nearly 40 % and lead to improved dyspnoea scores and lung function; on the other hand, corticosteroids enhance the apoptosis of Th2 cells, and subsequently reduce natural immunity thereby leading to hyper-infection/disseminated infection [16, 17]. It has been proposed that corticosteroids increase ecdysteroid-like substances (naturally occurring sterols with non-hormonal anabolic effects) which act as moulting signals causing the rhabtidiform larvae to change into infective filariform larvae which amply the infection, eventually leading to hyper-infection syndrome [10]. The pulmonary symptoms are a consequence of the organism's normal life cycle, in which the filariform larvae progress to the lungs and are swallowed. On entry into the intestine, the filariform larvae mature into adult females and produce rhabtidiform larvae that mature into infectious filariforms, either in the intestine or in the environment [18].

Therefore, all those those who harbour *S. stercoralis* should be treated, even if they are asymptomatic, to curtail the risk of the hyper-infection syndrome [7]. The diverse clinical manifestations of strongyloidosis are rarely associated with diagnosis until it is too late to successfully treat the infection, and the pulmonary symptoms are frequently regarded as an exacerbation of COPD [19]. This leads to the initiation or an increase in steroid therapy, which leads to more rapid progression of the strongloides hyper-infection [20]. In turn, this results in severe complications that are often fatal [12]. Mortality approaches nearly 90 % in immunocompromised patients receiving corticosteroid therapy [21]. A high index of suspicion is important to protect those with who are receiving steroid therapy and those immunocompromised by viruses such human T-lymphotropic virus (HTLV-1) and HIV, those receiving immunosuppressive therapy, patients with haematological malignancies, and those with diabetes and malnutrition [17, 22].

The diagnosis of strongyloidiasis is based on the observation of juvenile larvae in copro-parasitological studies [20]. The gold standard for the diagnosis of strongyloidiasis is serial examination of the parasites with routine saline or wet mount preparations, concentration techniques (Baermann concentration, Horadi–Mori filter paper culture, quantitative acetate concentration technique), culturing the samples on agar plates (faecal, sputum, BAL, duodenal aspirate), and histopathological and cytological studies (duodenal biopsy, duodenal aspirate) [10, 23].

Numerous larvae of *S. stercoralis* were found in the patient’s BAL and faeces; and vancomycin-sensitive *Enterococcus* species from blood samples provided diagnostic proof of strongyloides hyper-infection syndrome with enterococcal sepsis, relating to our case [24]. However, it is widely reported that eosinophils may be decreased during hyper-infection and this may be an explanation of the poor prognosis. We did not determine whether the patient had pulmonary eosinophilia, which is often seen as larvae of *S. stercoralis* pass through the lung [25, 26].

Ivermectin and benzimidazoles (thiabendazole, mebendazole and albendazole) are becoming the drugs of choice for strongyloides infection in many countries [27, 28]. Ivermectin inhibits neurotransmission, while benzimidazoles disrupt energy production in the parasites with optimal anthelmintic activities [7]. However, early detection and subsequent dosing schedules, plus monitoring for toxicity of ivermectin and albendazole is mandatory [27]. Additionally, post-therapy stool examinations are also recommended to verify *Strongyloides* eradication and to exclude other parasitic infections.

Broad-spectrum antibiotic therapy (directed toward enteric pathogens), supportive treatment (intravenous fluids if volume depletion, blood transfusion if gastrointestinal or alveolar haemorrhage, mechanical ventilation if respiratory failure) and symptomatic treatment are crucial in case management [1, 7]. Meanwhile, empirical steroid therapy is contraindicated because of its immunosuppressive effects, which increase susceptibility to parasitic infections and the apparent role of this therapy as a maturation factor causing the rhabtidiform larvae to mature into infectious filariform larvae, amplifying the infection [13, 22].

To the best of our knowledge, this is the first case report of strongyloidiasis hyper-infection with concurrent enteric sepsis from Nepal. Despite intensive care, aggressive antibiotics and anti-helminthic therapy, the patient died. Diagnostic delay, empirical steroid therapy and enterococcal bloodstream infection – caused by the filariform larvae moving from the colon to the bloodstream – were the probable causes of death in this case [21, 29].

### Conclusion

Avoiding corticosteriod therapy in strongyloidiasis is imperative and thus a high index of suspicion is required. An early diagnosis followed by prompt administration of anti-helminthic therapy is required to eradicate this infection before the infected patient is subjected to immunosuppressive therapy.

## Supplementary Data

Supplementary File 1

Supplementary File 2
