# Supplementary material for: Fatal strongyloidiasis after corticosteroid therapy for presumed chronic obstructive pulmonary disease
Source: JMM Case Rep. 2018 Sep 11;5(9):e005165. doi: 10.1099/jmmcr.0.005165 (PMC6230759; doi:10.1099/jmmcr.0.005165)
Supplement: Supplementary File 1 [file jmmcr-5-5165-s001.pdf]

Fatal Strongyloidiasis after empiric corticosteroid therapy.

Earlier 3 months

- past historyof chronic obstructive pulmonary disease, systemic hypertension, and previously treated for pulmonary tuberculosis.
- the patient was under oral steroid (Prednisolone 60 mg/gl) 3 months ago tapered to 5mg OD with symptomatic improvement

3rd November,2016

- Hospitalized in a local hospital with a diagnosis of acute gastritis with acute exacerbation, COPD for two days .Hence was given intravenous ceftriaxone 2 gm once daily, azithromycin 500 mg once daily, methylprednisolone 40 mg three times daily, and salbutamol and ipratropium bromide nebulizer

15th November, 2016

- presented with chief complaints of nausea, vomiting, joint pain ,crampy abdominal pain admitted in the emergency ICU,Sumeru Hospital.
- found hypotensive and hypoxemic with body temperature 100˚F, blood pressure 78/35 mmHg, arterial partial pressure of oxygen (PaO2)69mmHg
- Preliminary diagnosis of septic shock from abdominal source, and acute respiratory failurewas made.Mechanical ventilation, aggressive volume resuscitation, and vasopressor support were given. Plus, Piperacillin/ tazobactam was administered for empirical anti-infection treatment
- Radiological assessment with Chest X-ray, showed collapse consolidation with pleural effusion on right lower lobe, hilar lymphnodes and cardiomegaly

17th November ,2016

- Bronchoscopy showed severe diffuse alveolar hemorrhage .
- Filariform larvae of *Strongyloides stercoralis* was seen on AFB preparation of BAL
- stool specimen revealed the numerous larvae of *Strongyloides stercoralis*
- Vancomycin sensitive *Enterococcus faecium* isolated from blood sample
- Treated with Ivermectin and Albendazole for Strongyloides; Linezolid plus Vancomycin were prescribed for two different strains of *E. faecium*

19th November,2016

- C-reactive protein doubled to 220mg/L, WBC count reduced to 2.9 ×109 cells/L, hemoglobin concentration reduced to 48 g/L,platelet count squeezed to 7×109 cells/L, total bilirubin increased to 103 mmol/L

21th November 2016

- patient developed a high grade fever, vomiting, lower abdominal pain, abdominal distention and constipation, dyspnea, wheezing and pleuric pain and acute respiratory distress syndrome (ARDS), mechanically ventilated,patient succumbed to his illness on 6th day of admission

Fig.S1 – Timeline.

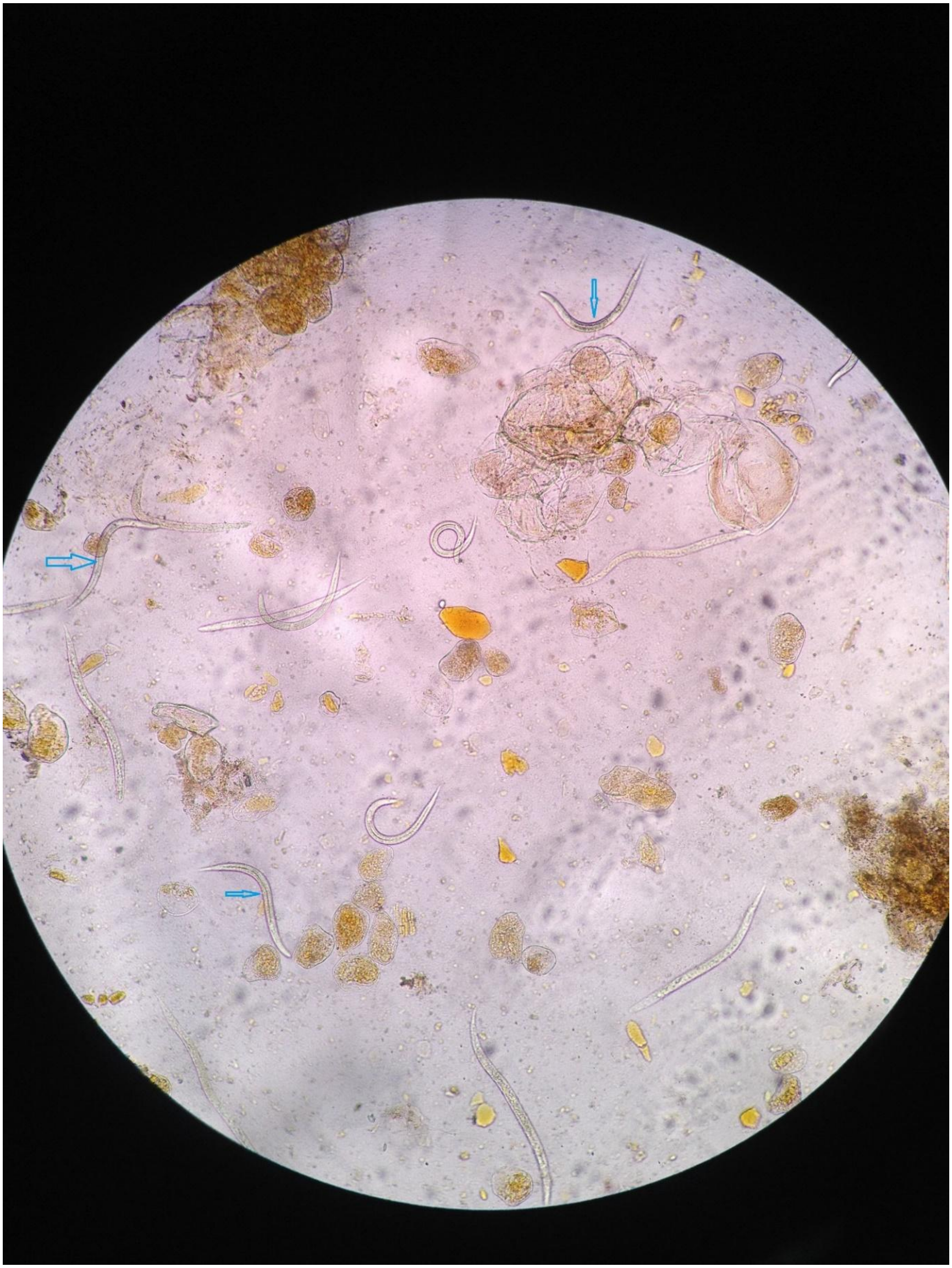

Fig. S2-Wet mount: Numerous rhabditiform larva of *Strongloides stercoralis* in an unstained wet mount of stool.
